# Dual-functional alginate and collagen–based injectable hydrogel for the treatment of cancer and its metastasis

**DOI:** 10.1186/s12951-022-01458-x

**Published:** 2022-05-28

**Authors:** Juyoung Hwang, Eun-Koung An, Wei Zhang, Hyo Jeong Kim, Youngho Eom, Jun-O. Jin

**Affiliations:** 1 Shanghai Public Health Clinical Center & Institutes of Biomedical Sciences, Shanghai Medical College, Fudan University, Shanghai, 201508 China; 2 Department of Medical Biotechnology, Yeungnam University, Gyeongsan, 38541 Republic of Korea; 3 Research Institute of Cell Culture, Yeungnam University, Gyeongsan, 38541 Republic of Korea; 4 Department of Polymer Engineering, Pukyong National University, Busan, 48513 Republic of Korea

**Keywords:** Thermally responsive gel, Photothermal therapy, Immunotherapy, Cancer metastasis, Tumor recurrence

## Abstract

**Background:**

Immunotherapies have been gaining attention for the prevention of cancer recurrence and metastasis. Cancer immunotherapy can induce memory cells to target cancer-specific antigens and, thus, selectively kill cancer cells. However, there are difficulties in inducing cancer antigen–specific immunity due to limited knowledge regarding cancer antigens. In this study, we synthesized a dual-functional hydrogel to induce antigen generation and immune activation.

**Results:**

To elicit a cancer self-antigen–specific immune response, we synthesized an alginate-collagen–based injectable hydrogel, called thermally responsive hydrogel (pTRG), which was incorporated with indocyanine green and the immune stimulator polyinosinic:polycytidylic acid (poly I:C). pTRG was evaluated for its anticancer and anti-metastatic effects against CT-26 carcinoma and 4T1 breast tumor in mice by combining photothermal therapy (PTT) and immunotherapy. Near-infrared (NIR) irradiation promoted temperature elevation in pTRG, consequently exerting a therapeutic effect on mouse tumors. Lung metastasis was prevented in cured CT-26 tumor-injected mice following pTRG treatment via cancer antigen–specific T cell immunity. Moreover, pTRG successfully eliminated the original tumor in 4T1 tumor-bearing mice via PTT and protected them from lung metastasis. To further evaluate the carrier function of TRGs, different types of immunotherapeutic molecules were incorporated into TRGs, which led to the effective elimination of the first CT-26 tumor and the prevention of lung metastasis.

**Conclusions:**

Our data demonstrate that TRG is a efficient material not only for treating primary tumors but also for preventing metastasis and recurrence.

**Supplementary Information:**

The online version contains supplementary material available at 10.1186/s12951-022-01458-x.

## Background

Combination therapy has been promising in preventing cancer recurrence and metastasis [1, 2]. Previously, effective cancer treatment has involved the combination of chemotherapy, immunotherapy, radiation therapy, gene therapy, photodynamic therapy, and photothermal therapy (PTT) [3, 4]. Various nanostructures and carriers have been investigated for the delivery of these combination therapies [5, 6]. Among them, hydrogels have shown positive results owing to their low cytotoxicity, biodegradability, and ability to transport therapeutic materials [7, 8].

PTT has recently received tremendous attention, as it can mediate its therapeutic effects by exclusively increasing the temperature in the laser-irradiated area [5]. Generation of thermal energy from near-infrared (NIR) light requires an NIR absorber [9]. Delivery of an NIR absorber to tumor cells is the first step in PTT [10]. The targeted delivery of an absorber using carriers induces NIR irradiation–mediated apoptosis (programmed cell death) of specific cancer cells, consequently reducing the damage to healthy tissues [11]. Indocyanine green (ICG) is an NIR absorber that has been approved as an imaging agent for human use by the United States Food and Drug Administration and the European Medicine Agency [12, 13]. In addition to its function as an imaging agent, ICG exerts a thermal-responsive effect from NIR laser irradiation and is frequently used in PTT [5]. However, ICG is limited in its use as a photosensitizer due to its fast degradation and poor stability in the body [14]. Therefore, various nanomaterials have been evaluated for their efficacy in overcoming the limitations of ICG [15, 16].

Immunotherapy against cancer involves the activation of T lymphocytes [17]. Antigen-specific cytotoxic T lymphocytes and helper T cells selectively kill antigen-expressing cancer cells and prevent cancer recurrence and metastasis [18]. Antigen-specific T cell activation is mediated by antigen-presenting cells such as dendritic cells (DCs) and macrophages [19]. After antigen stimulation, DCs upregulate the expression of co-stimulators and present antigens on the major histocompatibility complex (MHC) [20]. In addition, activated DCs secrete pro-inflammatory cytokines that induce T cell differentiation and activation. However, the antigens generated by cancer cells are non-immunogenic, and thus, can neither promote sufficient DC activation nor mediate their presentation [20]. Cancer cell apoptosis also produces cancer antigens that do not fully activate DCs [21]. Therefore, immunostimulatory molecules and adjuvants are commonly included in therapeutic trials for effective cancer immunotherapy [22].

In mice, conventional DCs (cDCs) comprise two subsets, namely, cDC1 and cDC2. cDC1 presents intracellular antigens to CD8 T cells via the MHC class I, whereas cDC2 promotes CD4 T cell activation by presenting extracellular antigens via the MHC class II [23]. Although activation of both CD4 and CD8 T cells is essential for cancer immunotherapy, anticancer immunity is attributed to activated CD8 T cells, which are also called cytotoxic T lymphocytes (CTLs) [24]. Antigen-specific CTL activation elicits the selective killing of cancer cells based on the expression of their antigens. Therefore, antigen-specific CTL activation is a desirable strategy for cancer immunotherapy [19].

Various types of hydrogels have been developed for cancer therapy, including injectable and attachable types [10, 25]. Hydrogels offer great advantages as carriers and can easily deliver anticancer drugs, photosensitizers, and immune stimulators [7, 26]. Given their excellent biocompatibility and biodegradability, polysaccharide-based hydrogels have been used to control drug delivery for cancers [27, 28]. Among the various types of polysaccharides, alginate (a natural biopolymer) has been frequently used for the synthesis of hydrogels because of its minimal toxicity, good biocompatibility, and biodegradability [29, 30]. Collagen, an abundant protein in animals and a major component of the connective tissues, forms a triple helix [31, 32]. The cross-linking of the molecular structure of collagen biopolymers with alginate, oxazolidine, or hyaluronic acid can increase its mechanical strength [31–33]. In this study, we fabricated a thermally responsive hydrogel (TRG) using sodium alginate–crosslinked collagen and incorporated ICG as the photosensitizer. TRG was also incorporated with polyinosinic:polycytidylic acid (poly I:C), termed pTRG, to activate an immune response. We evaluated whether pTRG could eliminate the tumor via PTT and further prevent cancer recurrence or metastasis by mediating cancer antigen–specific immunity.

## Materials and methods

### Preparation of pTRG

The TRG was formulated from alginate/collagen, as described in a previous study [34–36]. Briefly, sodium alginate (Sigma Aldrich, St. Louis, MO, USA) was dissolved in deionized water (DW) at a concentration of 40 mg/mL. Collagen (from calf skin, Sigma Aldrich) was dissolved in 0.1 M acetic acid at a concentration of 60 mg/mL. The alginate solution (40 mg/mL) was mixed with collagen solution (60 mg/mL) at a 1:1 volumetric ratio. To prepare the TRG, ICG (Tokyo Chemical Industry Co., Ltd., Tokyo, Japan), along with a 5% weight ratio of collagen, was dissolved in DW and added to the solution. The pre-gel solution was treated with 150 mM calcium chloride (CaCl_2_; Sigma Aldrich). For the evaluation of the carrier effect of TRGs, the TRG was incorporated with 0.8 mg/mL of poly I:C (HMW, InvivoGen, San Diego, CA), 1.8 mg/mL of stimulator of interferon genes (STING) ligand (2′3'-cGAMP, InvivoGen), 6.3 mg/mL of anti-programmed cell death-1(PD-1) antibody (Ab) (29F.1A, BioXcell, Lebanon, NH, USA), or 4.4 mg/mL of the anti-programmed cell death-ligand 1 (PD-L1) Ab (29F1A12, BioXcell), respectively.

### Characterization of pTRG

Scanning electron microscopy (SEM) images were obtained using an S-4800 scanning electron microscope (HITACHI, Japan). Released poly I:C, STING ligand, anti-PD-1 Ab, and anti-PD-L1 Ab from TRG after 808-nm laser irradiation were quantified by obtaining the difference between the total quantity and the supernatant quantity. To determine the concentration of poly I:C released from the TRG, absorbance using a UV/vis spectrophotometer (Cary 100 Bio, Varian Inc., Palo Alto, CA, USA) was measured at 280 nm. The released concentration of STING ligand was measured using the 2′3'-cGAMP enzyme-linked immunosorbent assay (ELISA) kit (Cayman Chemical Company, Ann Arbor, MI, USA). In addition, the concentrations of the released anti-PD-1 and anti-PD-L1 Abs were determined using indirect ELISA. The recombinant murine PD-1 or PD-L1 were precoated in the plate; then, we added the released Ab-containing buffer. After washing with PBS, HRP-conjugated secondary Abs were added, and the concentration of the released Abs was determined using a UV/vis spectrophotometer (Cary 100 Bio, Varian Inc.). A fiber-coupled continuous-wave diode laser (808 nm, 10 W) was obtained from Changchun New Industries Optoelectronic Technology Co., Ltd. (China). Thermographic images were obtained using the FLIR One Thermal Imaging System (FLIR Systems, Wilsonville, OR, USA). Rheological properties of TRG were characterized using a rotational rheometer (MCR 302, Anton Paar, Austria). A 4.7-mL-volume coaxial cylinder (CC17) was used for the rheological measurement. The dynamic frequency sweeping was carried out at 25 °C at a 5% strain level over an angular frequency range of 0.05–200 rad s^−1^. The thermally responsive gel-sol transition was assessed by the temperature sweeping test. The heating measurement was conducted at an angular frequency and strain level of 1 rad s^−1^ and 5%, respectively, and the temperature was ramped at a heating rate of 5 °C min^−1^ over the range of 25–70 °C.

### Cell lines

The murine colon carcinoma cell line CT-26-iRFP (CT26.WT-iRFP-Neo; Imanis Life Sciences, CL091, Rochester, USA) was cultured in Dulbecco’s modified Eagle’s medium (Thermo Fisher Scientific, Inc., Waltham, MA, USA) supplemented with 10% fetal bovine serum (FBS, Sigma Aldrich), 1% penicillin/streptomycin (Gibco BRL Ltd, Paisley, Scotland), and 0.4 mg/mL G418 (Thermo Fisher Scientific, Inc.). The murine breast cancer cell line 4T1-iRFP (4T1.WT-iRFP-Neo; Imanis Life Sciences, CL078) was cultured in Roswell Park Memorial Institute medium (Thermo Fisher Scientific, Inc.), supplemented with 10% FBS (Sigma Aldrich), 1% penicillin/streptomycin (Gibco BRL Ltd, Paisley, Scotland), 0.1 mg/mL G418 (Thermo Fisher Scientific, Inc.), and 2 μg/mL puromycin (InvivoGen). The cell lines were cultured at 37 °C in a humidified atmosphere containing 5% CO_2_ and air.

### Mice

BALB/c mice (6–8 weeks old, female) were obtained from Hyochang Science (Daegu, Republic of Korea). The mice were maintained under pathogen-free conditions at the Laboratory Animals Center of Yeungnam University. All experiments were conducted in consideration of the basic ethical principles of animal experiments and the 3R principles. In addition, the experiments were conducted in compliance with the animal protection law, the law on experimental animals, and the IACUC regulations of Yeungnam University. The Committee on the Ethics of Animal Experiments of Yeungnam University Laboratory Animals Center approved the protocol (mouse protocol number, 2020–039). For ethical reasons, we minimized the pain or stress experienced by the animals by euthanizing them with CO_2_ gas, following the humanitarian endpoint criteria.

### First tumor challenge and PTT

BALB/c mice were subcutaneously injected with 1 × 10^6^ CT-26-iRFP or 4T1-iRFP cells. After 7 days, the mice were randomly distributed into six groups as follows: PBS, poly I:C, Gel, pGel, TRG, and pTRG. After an intratumoral (*i.t.*) injection of 50 µL of the sample using a 21-gauge needle, the tumor was irradiated with an 808-nm laser at 1.5 W/cm^2^ for 5 min. The elevated temperature was imaged using the FLIR One Thermal Imaging System (FLIR Systems). The tumor volume was monitored on the 28th day after the tumor challenge and calculated using the formula V = ½ (length × width^2^).

### Antibodies

Anti-IgG1, anti-IgG2a, and anti-IgG2b Abs were used as isotype controls and purchased from BioLegend (San Diego, CA, USA). Allophycocyanin (APC)-Cy7-anti-T cell receptor (TCR)-β (H57-597), PerCP5.5-anti-CD4 (GK1.5), BV785-anti-CD8α (53–6.7), phycoerythrin (PE)/cy7-anti-interferon-gamma (IFN-γ) (B27), BV 785-anti-CD11c (N418), Alexa Fluor 647-anti-CD40 (HM40-3), BV 605-anti-CD80 (16-10A1), PE/Cy7-anti-CD86 (GL-1), PerCP5.5-anti-MHC class I (AF6-88.5), and PE-anti-MHC class II (M5/114.15.2) were obtained from BioLegend.

### Mouse lymphoid DC analysis

Tumor-draining lymph nodes (tdLNs) were homogenized using a glass slide. Lipids and debris were removed using a 100-nm nylon mash. After washing with PBS, the cells were stained with fluorescence-labeled Abs for 30 min. To define DCs, the cells were stained with lineage markers, including anti-B220 (RA3-6B2), anti-CD3 (17A2), anti-CD49b (DX5), anti-Gr1 (RB68C5), anti-Thy1.1 (OX-7), and anti-TER-119 (TER-119). Lineage^–^CD11c^+^ cells were further divided into cDC1 and cDC2 cells. Co-stimulator and MHC molecule levels were measured in cDC1 and cDC2 using a NovoCyte flow cytometer (ACEA Biosciences Inc., San Diego, CA, USA).

### Flow cytometry analysis

The tdLN cells were stained with unlabeled isotype control Abs and Fc-block Abs (BioLegend) for 15 min to prevent any non-specific binding. After washing with PBS, the cells were stained with fluorescence-conjugated Abs at 4 °C for 30 min. After removing the free Abs by washing with PBS, the cells were suspended in a flow cytometry buffer (BioLegend) containing 4',6-diamidino-2-phenylindole (Sigma Aldrich) and were analyzed using a NovoCyte flow cytometer (ACEA Biosciences Inc.).

### Second tumor challenge

The mice cured of the CT-26 or 4T1 tumor were intravenously (*i.v.*) injected with CT-26-iRFP (0.7 × 10^6^/100 µL of PBS) or 4T1-iRFP (0.5 × 10^6^/100 µL of PBS) cells, respectively. On the 10th day after the second cancer challenge, the mice were euthanized, and their lungs and spleen were harvested for further experiments.

### In vivo fluorescence imaging

iRFP fluorescence images were captured using the fluorescence in vivo imaging system, FOBI (Cellgentek, Cheongju, Republic of Korea) after the first and second challenges.

### Histology

As previously described in detail, lung samples were harvested 10 days after the second tumor challenge and fixed with 10% formalin. After rehydration with ethanol gradient solutions, the lungs were embedded in paraffin and sliced into 5-μm thick sections using a microtome. The sections were attached to a glass slide, rehydrated, and stained with hematoxylin and eosin. Any tumor infiltration in the lung was observed using an EVOS M5000 microscope (Invitrogen, Waltham, MA, USA).

### Analysis of CT-26–specific T cell immunity

CT-26 cells (1 × 10^7^ CT-26) were freeze-dried and thawed thrice to obtain a lysate. After centrifugation (10,000*g* at 4 °C for 5 min), the protein concentration of the CT-26 supernatant was determined using the Bradford assay. Splenocytes (1 × 10^6^ splenocytes) were treated with 10 μg/mL CT-26 cell lysate. Twenty-four hours after incubation, the cells were stained with surface Abs (APC/Cy7-anti-TCR-β, PerCP5.5-anti-CD4, and BV785-anti-CD8α) for 20 min. After washing with PBS, the cells were fixed and permeabilized with an intracellular staining buffer (BioLegend) at 4 °C for 30 min. After washing, the cells were incubated with intracellular Abs (PE-conjugated anti-IFN-γ) for 30 min. IFN-γ-producing CD4 and CD8 T cells were analyzed using NovoCyte (ACEA Biosciences Inc.).

#### ELISA

Serum concentrations of interleukin (IL)-6, IL-12p70, and tumor necrosis factor (TNF)-α were measured 24 h after the first tumor therapy using ELISA kits (BioLegend). Antigen-specific production of TNF-α, IFN-γ, perforin, and granzyme B in the cultured medium was analyzed 24 h after stimulation of splenocytes with 10 μg/mL of CT-26 lysate.

### In vivo cytotoxicity assay

Splenocytes were harvested from BALB/c mice and labeled with 200 nM of carboxyfluorescein succinimidyl ester (CFSE) or 10 mM CellTracker™ Orange CMTMR (Life Technologies). CFSE-labeled splenocytes were coated with 200 nM of CT-26 antigen AH1 (SPSYVYHQF) peptide, and the CMTMR-labeled cells were loaded with the control peptide. CFSE and CMTMR-labeled cells were mixed at a 1:1 ratio, and a total of 10 × 10^6^ labeled cells were transferred to BALB/c mice, which were treated with TRG and pTRG for the first time. The mice treated with PBS, poly I:C and pGel were also exposed to labeled cells as controls. Twelve hours after the splenocyte transfer, the spleen was harvested, and specific killing was analyzed using NovoCyte (ACEA Biosciences Inc.).

### Depletion of CD4 and CD8 T cells

After treatment of the first tumor by PTT, the cured mice received 1 mg/kg of anti-CD4 Ab (GK1.5, 1) or 1 mg/kg of anti-CD8 Ab (YTS169.4) (both from BioXcell) every 2 days from the 28th day after the first tumor challenge (2 days before the second challenge of cancer cells). The depletion efficacy of Ab injection was analyzed using NovoCyte (ACEA Biosciences Inc.); we observed > 98% depletion of CD4 or CD8 cells in the mice.

### Statistical analysis

Data were analyzed using SPSS (Chicago, IL, USA) and expressed as the mean ± standard error. The values *p < *0.05,* and **p < *0.01* were considered statistically significant.

## Results

### Preparation and characterization of TRG

**Fig. 1:**
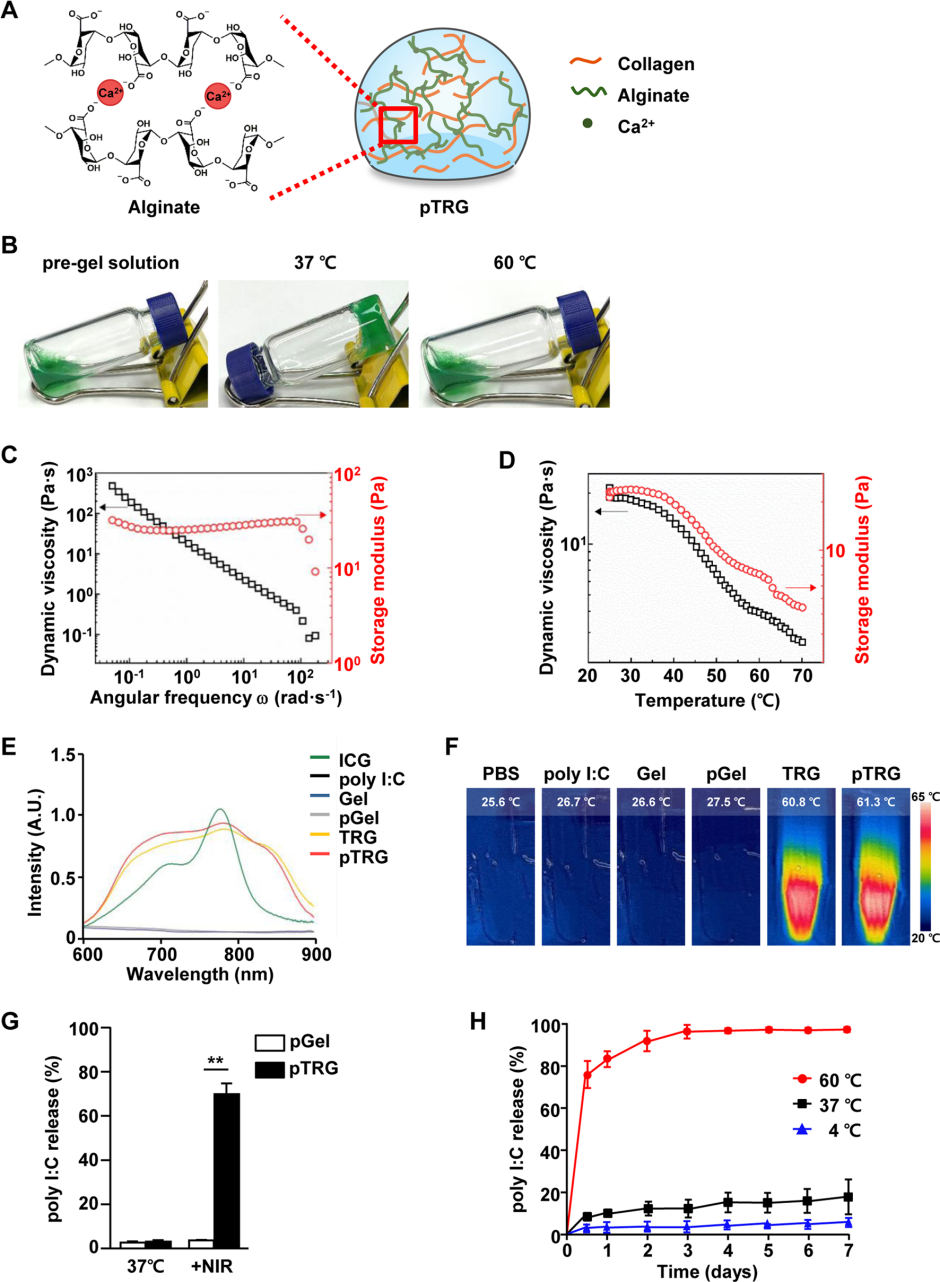
Characterization of the thermally responsive hydrogel (TRG). **A** Schematic illustration of alginate gelation secondary to the ionic interaction with Ca^2+^. **B** Photographs of TRG at different temperatures. **C** Dynamic viscosity (*η'*) and storage modulus (*G'*) curves of TRG at 25 ℃ over an angular frequency range of 0.05–200 rad s^−1^. **D** Variations of dynamic viscosity (*η'*) and storage modulus (*G'*) for TRG with increasing temperature at a heating rate of 5 ℃ min^−1^ over a temperature range of 25–70 ℃. **E** UV–vis NIR absorption spectra of free ICG, poly I:C, Gel, poly I:C–containing gel (pGel), ICG-incorporated TRG, and poly I:C–containing TRG (pTRG). **F** Thermographic images of PBS, poly I:C, Gel, pGel, TRG, and pTRG after 5 min under NIR laser irradiation at a laser power density of 1.5 W/cm^2^. **G** Poly I:C released from pGel and pTRG in PBS at 37 ℃ or under NIR laser irradiation (1.5 W/cm^2^, 5 min; **p < *0.01*). **H** Cumulative release of poly I:C from pGel after laser irradiation (1.5 W/cm^2^, 5 min). The percentage of the poly I:C released in the supernatant at the indicated time points

The guluronate of alginate forms a 3D network in the presence of Ca^2+^ (Fig. 1A) [27]. Sodium alginate–crosslinked collagen was mixed with CaCl_2_ to form a hydrogel at 37 °C. At 60 °C, the gel changed into a fluid (Fig. 1B). The rheological properties and thermal-responsive phase transition of the hydrogel were evaluated using a dynamic frequency test. Dynamic viscosity (*η'*) and storage modulus (*G'*) curves of the hydrogel are plotted as a function of angular frequency in Fig. 1C. The sharp decreasing trend of *η'* is called the Bingham behavior, which is a typical phenomenon of heterogeneous systems, including hydrogels [37]. In other words, the shear-induced collapse of the gel structure is responsible for the marked viscosity reduction. The plateau *G'* curve is another evidence of the formation of the gel structure. The *G'* is a measure of elasticity of a polymer system [38]. In the case of hydrogels, the presence of a 3D gel network affords high elasticity even at low shears, which results in the plateau-like *G'* curve independent of angular frequency [38]. The thermal-induced changes in *η'* and *G'* are shown in Fig. 1D. Both rheological parameters exhibit sharp reduction above 40 °C, and the viscosity at 60 °C is much lower than that at 25 ℃, indicating the gel-sol transition. The TRG was further incorporated with poly I:C to obtain pTRG. As shown in Fig. 1E, TRG and pTRG showed maximum absorbances at 780 nm. For the evaluation of photothermal efficiency, the TRG and pTRG were irradiated with an NIR laser (1.5 W/cm^2^ for 5 min) that resulted in an efficient increase in the temperature to over 60 °C (Fig. 1F); the gel changed to fluid at 60 °C. Next, we examined whether poly I:C was released from pTRG after NIR irradiation and found that 69.5% of poly I:C (0.556 mg/mL) was released from pTRG after 5 min of NIR laser irradiation. In contrast, the poly I:C–containing gel (pGel, without ICG) did not release poly I:C (Fig. 1G). To determine the stability of pTRG in an aqueous solution, we maintained it in an FBS-containing culture medium at different temperatures for 7 days. The cumulative poly I:C released from pTRG was 98.7% (0.79 mg/mL), 19.8% (0.79 mg/mL), and 5.0% (0.04 mg/mL) at 60 °C, 37 °C, and 4 °C, respectively (Fig. 1H). Thus, these results indicate that pTRG functions as a photothermal material when exposed to an NIR laser.

### Evaluation of the photothermal therapeutic effect of the TRG and pTRG against the CT-26 tumor

**Fig. 2:**
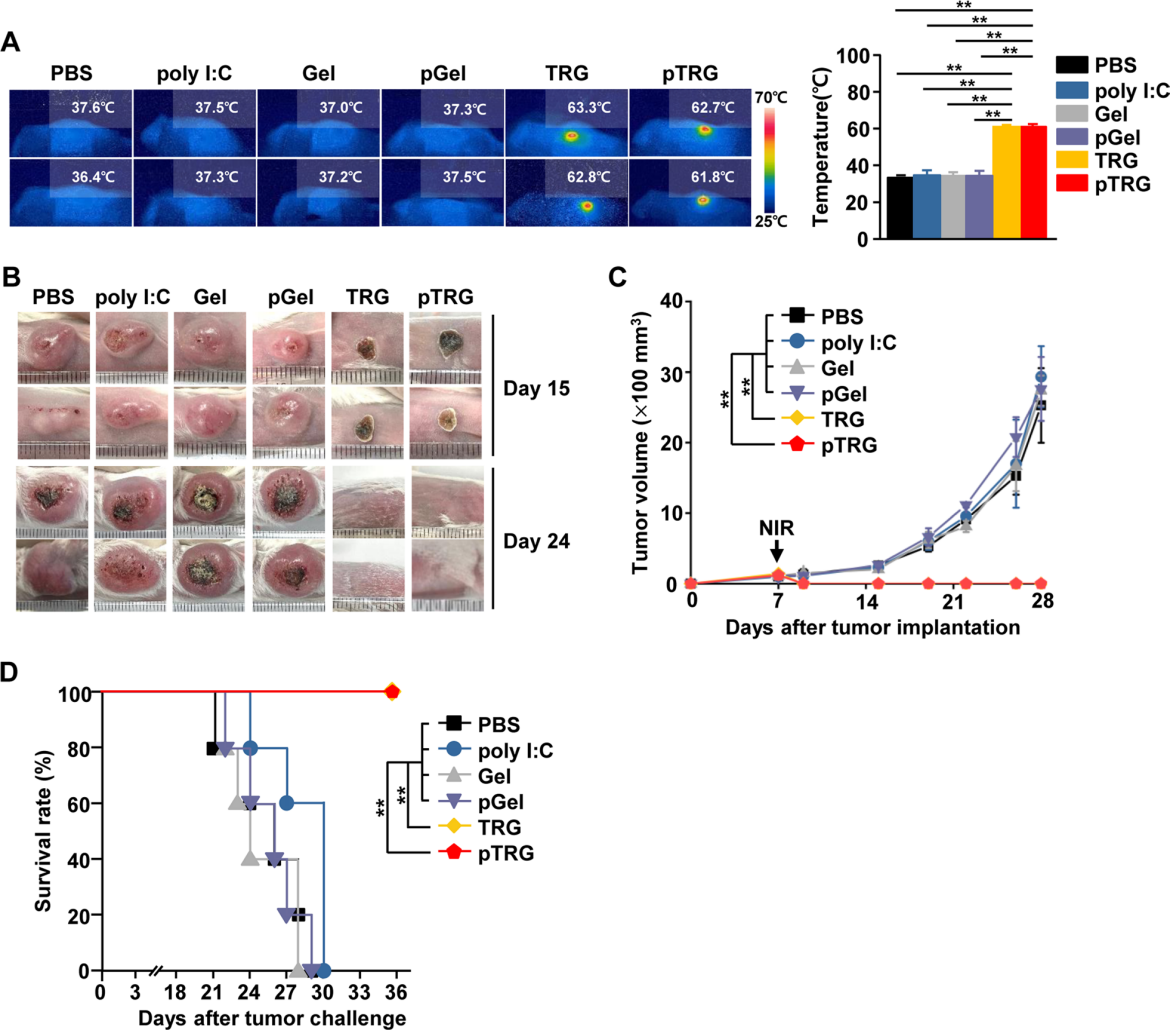
Photothermal therapy with a combination of pTRG and NIR laser irradiation protected the mice from the CT-26 tumor. BALB/c mice were subcutaneously (*s.c.*) injected with 1 × 10^6^ CT-26-iRFP cells. On the 7th day of tumor inoculation, the mice were intratumorally (*i.t.*) administered PBS, poly I:C, Gel, pGel, TRG, or pTRG and were then subjected to NIR laser irradiation for 5 min at a laser power density of 1.5 W/cm^2^. **A** Thermographic images of surface temperature at the tumor sites (left panel). Mean temperature after NIR laser irradiation (right panel, n = 6 mice, two-way ANOVA, mean ± SEM, **p < *0.01*). **B** CT-26 tumor masses on days 15 and 24 after tumor injection. **C** Tumor growth curves of CT-26 mice (n = 6 mice, significance was determined using the log-rank test, mean ± SEM **p < *0.01*). **D** Survival rates of mice (curve comparison with the log-rank test revealing statistically significant differences, **p < 0.01, n = 5 mice per group)

Based on the thermal-responsive effect of the hydrogel, we evaluated its photothermal therapeutic effect against CT-26 carcinoma in BALB/c mice. CT-26 tumor-bearing mice were injected *i.t.* with the hydrogels 7 days after tumor injection. Under a 5-min NIR laser irradiation (1.5 W/cm^2^), the temperatures of the TRG- and pTRG-injected tumors were greatly increased; the maximum temperatures of TRG and pTRG were 63.3 ℃ (± 0.49 ℃) and 62.7 ℃ (± 0.94 ℃), respectively (Fig. 2A). There were no considerable changes in the temperatures of PBS-, poly I:C-, Gel-, or pGel-treated tumors under NIR laser irradiation (Fig. 2A). Furthermore, tumors in the laser-irradiated mice that received TRG and pTRG showed substantial decreases in size and the remaining burn wound on day 15 after tumor injection (Fig. 2B). The tumor completely disappeared on day 24 after tumor injection (Fig. 2B). The other controls had large tumor masses (Fig. 2B). As seen in the tumor growth curve in Fig. 2C, TRG and pTRG with laser irradiation eliminated the CT-26 tumors, whereas the tumor volumes gradually increased in the other controls. Therefore, mice treated with TRG or pTRG along with NIR laser irradiation survived the CT-26 tumor challenge, whereas the other controls died within 27 days after CT-26 tumor injection (Fig. 2D). These data suggest that TRG and pTRG effectively eliminated CT-26 tumor growth through PTT.

### DCs in tdLNs were activated by pTRG and NIR laser irradiation

**Fig. 3:**
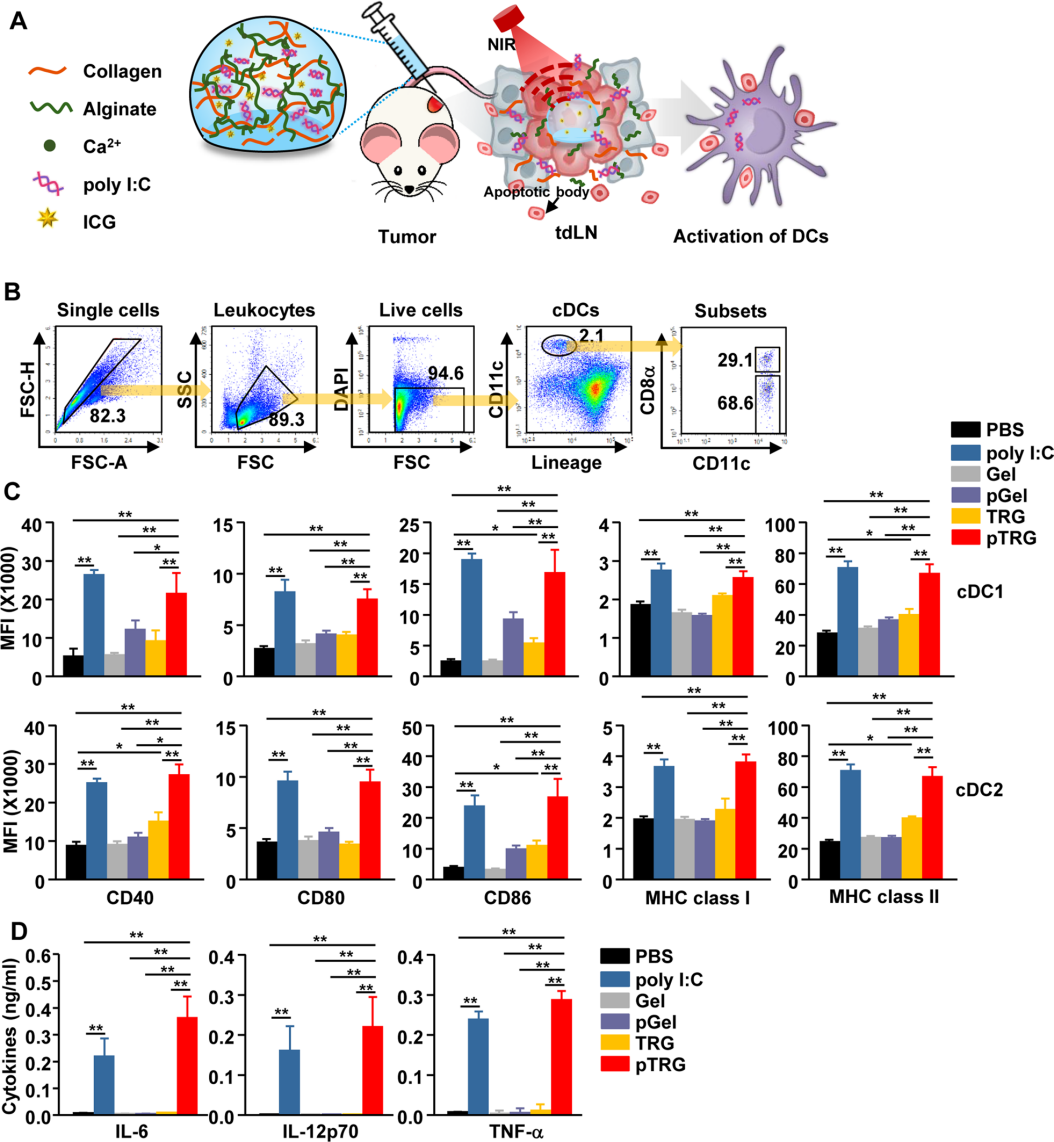
NIR laser irradiation of pTRG-treated tumors induced the activation of conventional dendritic cells (cDCs) in tumor-draining lymph nodes (tdLNs). **A** Schematic illustration of the analysis method for DC activation in tdLNs. **B** cDC subset analysis in tdLNs via flow cytometry. **C** The mean fluorescence intensities (MFIs) of CD40, CD80, CD86, MHC I, and MHC II in cDC1 (upper panel) and cDC2 (lower panel) were measured via flow cytometry (n = 6*,* two-way ANOVA, mean ± SEM, *p < 0.05, **p < *0.01*). **D** Serum concentrations of indicated cytokines were analyzed using enzyme-linked immunosorbent assays (ELISAs) (n = 6*,* two-way ANOVA, mean ± SEM, **p < *0.01*)

As high temperatures promote the release of poly I:C from the gel, we examined whether the released poly I:C induced the activation of DCs in tdLNs (Fig. 3A). CT-26 tumor-bearing mice were treated with hydrogels and irradiated with an NIR laser. Consistent with Fig. 2A, temperatures were elevated in TRG- and pTRG-treated tumors (Additional file 1: Figure S1). Twenty-four hours after NIR irradiation, tdLNs were harvested and the cDC subsets in tdLN cells were defined (Fig. 3B). The combination of pTRG and NIR laser irradiation induced a considerable upregulation in the expression of CD40, CD80, CD86, MHC class I, and MHC II in both cDC1 and cCD2, which was almost similar to that observed in the poly I:C treatment group (Fig. 3C). In addition, the serum concentrations of IL-6, IL-12p70, and TNF-α substantially increased after pTRG treatment with laser irradiation (Fig. 3D). In contrast with the pTRG treatment, Gel, pGel, and TRG treatments combined with laser irradiation did not activate the DCs or induce cytokine production (Fig. 3C, D). Therefore, these data suggest that pTRG and laser irradiation can induce the activation of DCs in tdLNs.

### Prevention of the second CT-26 tumor growth in the pTRG-cured mice

**Fig. 4:**
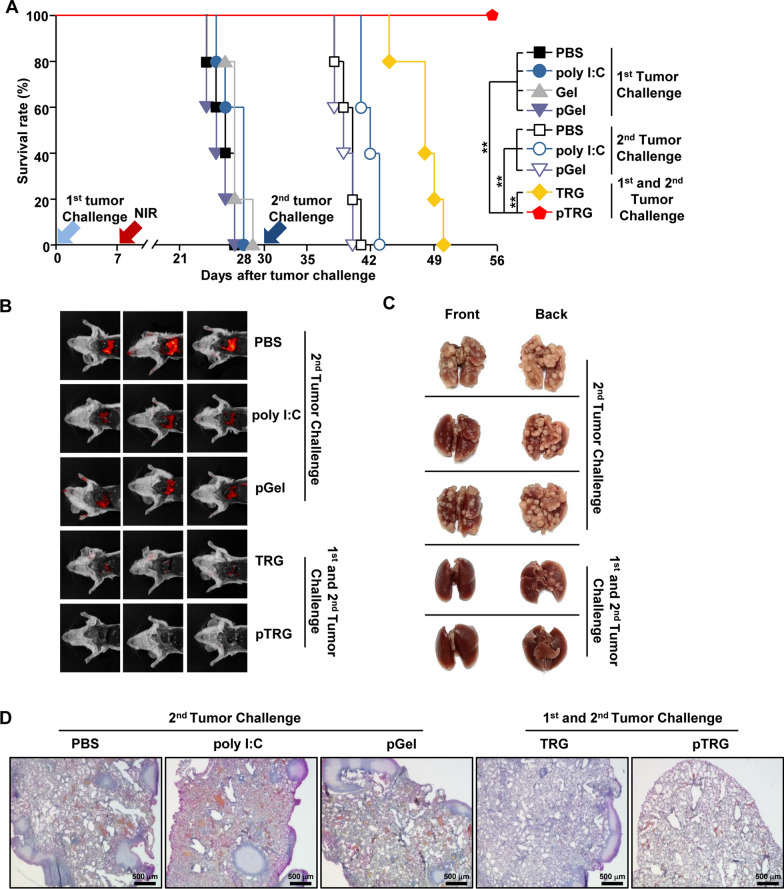
Preventive effect of pTRG from the second challenge of CT-26 cell growth in the lungs. After the first tumor therapy, the surviving mice were subjected to a second challenge with an intravenous injection of 0.7 × 10^6^ of CT-26-iRFP on the 30th day after the first tumor injection. **A** Survival rates of the mice were monitored (n = 5 per group). **B** Fluorescence images of CT-26-iRFP on day 10 after the second CT-26 challenge (n = 6). **C** Representative photographs of metastatic lung cancer (n = 6). **D** Tumor infiltration was analyzed via hematoxylin and eosin (**H**&**E**) staining of the lung tissue (n = 6)

Next, we examined the preventive effect of pTRG against the second tumor challenge. The schematic schedule of the first and second tumor challenges is shown in Additional file 1: Figure S2. The mice cured of the CT-26 tumor from the first challenge by the TRG and pTRG treatments were rechallenged with an *i.v.* injection of CT-26 cells (Additional file 1: Figure S2). In addition, PBS-, poly I:C-, and pGel-treated mice that were not challenged with the first tumor were injected *i.v.* with CT-26 cells (Additional file 1: Figure S2). As shown in Fig. 4A, PBS-, poly I:C-, and pGel-treated mice died within 13 days of tumor injection. TRG-cured mice from the first CT-26 injection showed delayed death and died within 20 days of the second CT-26 challenge (Fig. 4A). In contrast, pTRG-mediated cured mice from the first CT-26 challenge survived until day 26 after the second CT-26 challenge (Fig. 4A). Moreover, metastatic CT-26 tumor growth remarkably increased in the lungs on the 10th day after the second tumor challenge in mice treated with PBS, poly I:C, or pGel (Fig. 4B, C). Notably, pTRG completely prevented the second challenge-induced cancer growth in the lungs of mice cured of the first CT-26 challenge, whereas TRG-cured mice showed CT-26 tumor cell infiltration in the lungs (Fig. 4B, C). In the histological analysis, the infiltration of CT-26 tumor cells in the lungs was completely inhibited in pTRG-treated first tumor-cured mice, whereas TRG-cured mice from the first tumor challenge could not avoid CT-26 cell infiltration in the lungs (Fig. 4D). Therefore, these data suggest that pTRG-mediated first tumor therapy could prevent metastasis and recurrence of the tumor.

### pTRG induced antigen-specific T cell immunity

**Fig. 5:**
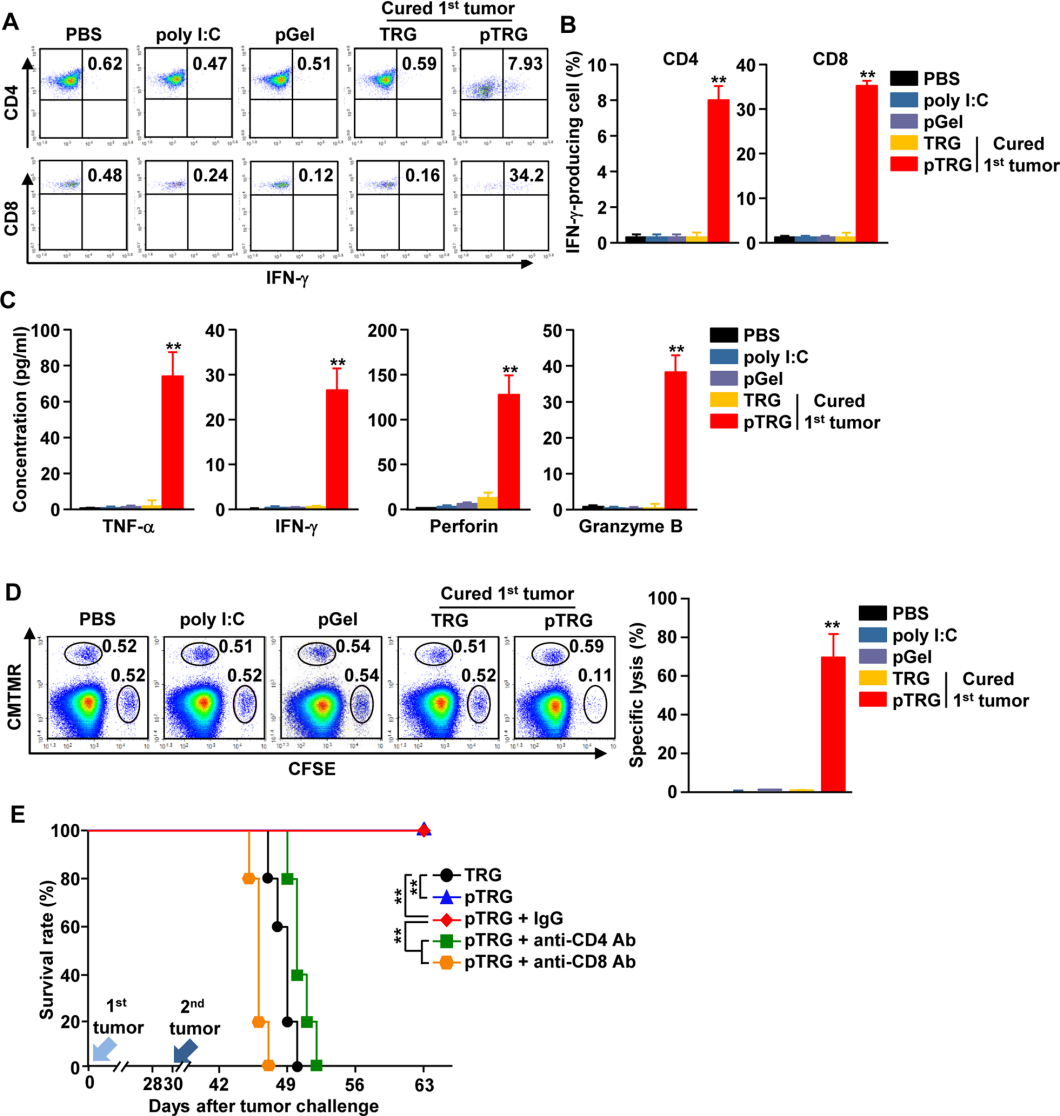
Antigen-specific T cell immunity induced by pTRG protected the mice from the second challenge with CT-26 cells. Splenocytes were harvested from mice on the 10th day after the second CT-26 challenge. Splenocytes (1 × 10^6^) were stimulated with 10 μg/mL of CT-26 cell lysate for 24 h. **A** Intracellular production of IFN-γ by CD4 and CD8 T cells. **B** Mean IFN-γ-producing cells among CD4 and CD8 T cells (n = 6*,* two-way ANOVA, mean ± SEM, **p < *0.01*). **C** Concentrations of indicated cytokines and cytotoxic mediators in culture media were measured via enzyme-linked immunosorbent assays (ELISAs) (n = 6*,* two-way ANOVA, mean ± SEM, **p < *0.01*). **D** In vivo CT-26 antigen-specific lysis was measured by CT-26 antigen-loaded splenocyte transfer. (n = 6*,* two-way ANOVA, mean ± SEM, **p < *0.01*). **E** The mice were injected with anti-CD4 and CD8 Abs every 2 days from the 28th day after the first tumor challenge. The survival rate of the mice is shown (n = 5 per group)

pTRG-mediated PTT induced the apoptosis of CT-26 cells expressing the CT-26 antigen. In addition, the poly I:C released from the pTRG promoted the activation of DCs in tdLNs. pTRG-treated mice were protected from the second tumor challenge. These results prompted us to examine whether pTRG treatment elicits CT-26 antigen-specific T cell immunity and whether T cells exert an anticancer effect against the second CT-26 challenge. On the 10th day after the second tumor challenge, the splenocytes were harvested and stimulated with CT-26 lysate. We observed a great increase in the intracellular production of IFN-γ in CD4 and CD8 T cells in pTRG-treated mice (Fig. 5A, B). However, the CD4 and CD8 T cells in the first tumor-cured TRG-treated mice and other controls did not produce IFN-γ in response to CT-26 lysate exposure (Fig. 5A, B). In addition, the concentrations of TNF-α, IFN-γ, perforin, and granzyme B were significantly higher in pTRG-treated splenocytes than in other treatment groups following CT-26 lysate exposure (Fig. 5C). Moreover, the pTRG-treated mice that were cured of the first CT-26 challenge showed a specific killing of the CT-26 antigen-coated splenocytes (Fig. 5D). Further evaluation of the CD4 and CD8 T cell contribution in the prevention of the second CT-26 challenge revealed that the mice cured with pTRG were depleted of CD4 and CD8 cells; these CD4- and CD8-depleted mice were not protected against the second CT-26 challenge, as evidenced by the tumor growth in their lungs (Fig. 5E). Together, these observations suggest that the pTRG-induced first tumor therapy promotes cancer antigen–specific T cell immunity, which primarily contributes to the prevention of the second tumor challenge in mice.

### Preventive effect of pTRG on the secondary 4T1 tumor challenge

**Fig. 6:**
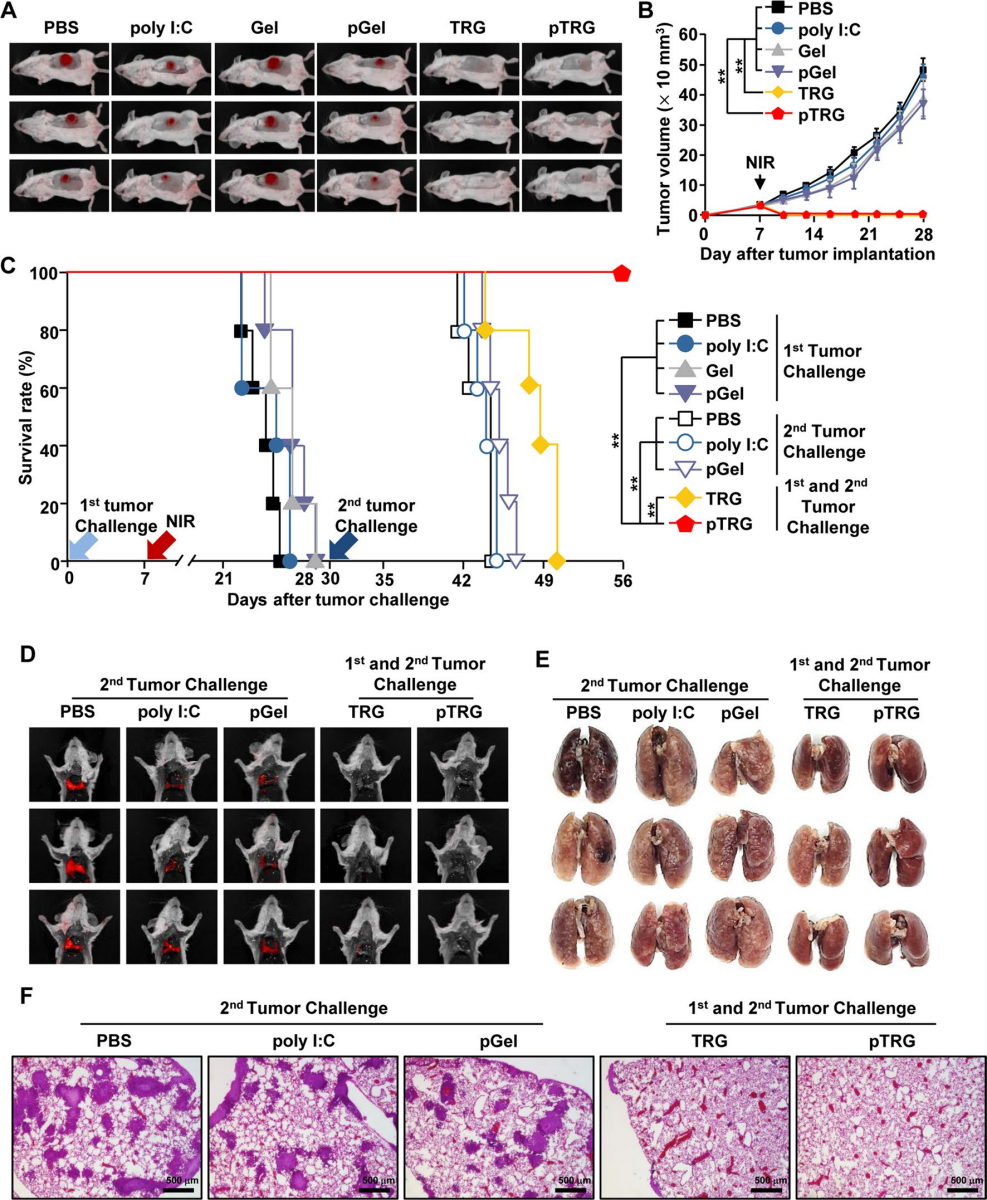
pTRG and NIR laser irradiation prevented 4T1 tumor growth. BALB/c mice were injected *s.c.* with 1 × 10^6^ 4T1-iRFP cells. On the 7th day after tumor implantation, the mice were *i.t.* treated with PBS, poly I:C, Gel, pGel, TRG, and pTRG and then subjected to NIR laser irradiation (1.5 W/cm^2^, 5 min). **A** iRFP imaging in 4T1 tumor-bearing mice on day 20 (n = 6). **B** Tumor growth curves of 4T1 mice (n = 6, significance was determined by the log-rank test, mean ± SEM **p < 0.01). **C**–**F** The surviving mice from the first 4T1 challenge were subjected to a second challenge with 4T1 cells (0.5 × 10^6^) by *i.v.* injection on the 30th day following the first tumor injection. **C** The mouse survival rates were monitored (n = 5 per group). **D** iRFP fluorescence images and **E** photographs of metastatic lung cancer on the 10th day after the second 4T1 challenge (n = 6). **F** Representative images of hematoxylin and eosin (**H**&**E**) staining of the lung tissues (n = 6)

Next, we examined whether pTRG can be used to treat a 4T1 tumor-bearing mice, a candidate tumor model for PTT, and the most well-defined metastatic murine cancer model. The 4T1 tumor-bearing BALB/c mice were injected *i.t.* with PBS, poly I:C, Gel, pGel, TRG, or pTRG and irradiated with NIR laser (Additional file 1: Figure S3). In mice treated with TRG and pTRG under NIR laser irradiation, the first 4T1 tumor was eliminated; the other control groups failed to eliminate the tumor (Fig. 6A, B). Consistent with the CT-26 tumor data, mice treated with pTRG and NIR laser irradiation survived the secondary challenge of metastatic lung cancer. TRG-treated mice and other control mice died within 20 days after the second challenge (Fig. 6C). Furthermore, the second challenge with 4T1 cells in pTRG-treated mice completely blocked the infiltration of 4T1 cancer cells into the lungs, whereas the TRG-cured mice from the first challenge failed to prevent 4T1 cell infiltration in the lungs (Fig. 6D–F). Therefore, these data suggest that pTRG can be used for the treatment of breast cancer via PTT and that it can prevent metastasis via immunotherapy.

### TRG as a therapeutic carrier to mediate anticancer effects

**Fig. 7:**
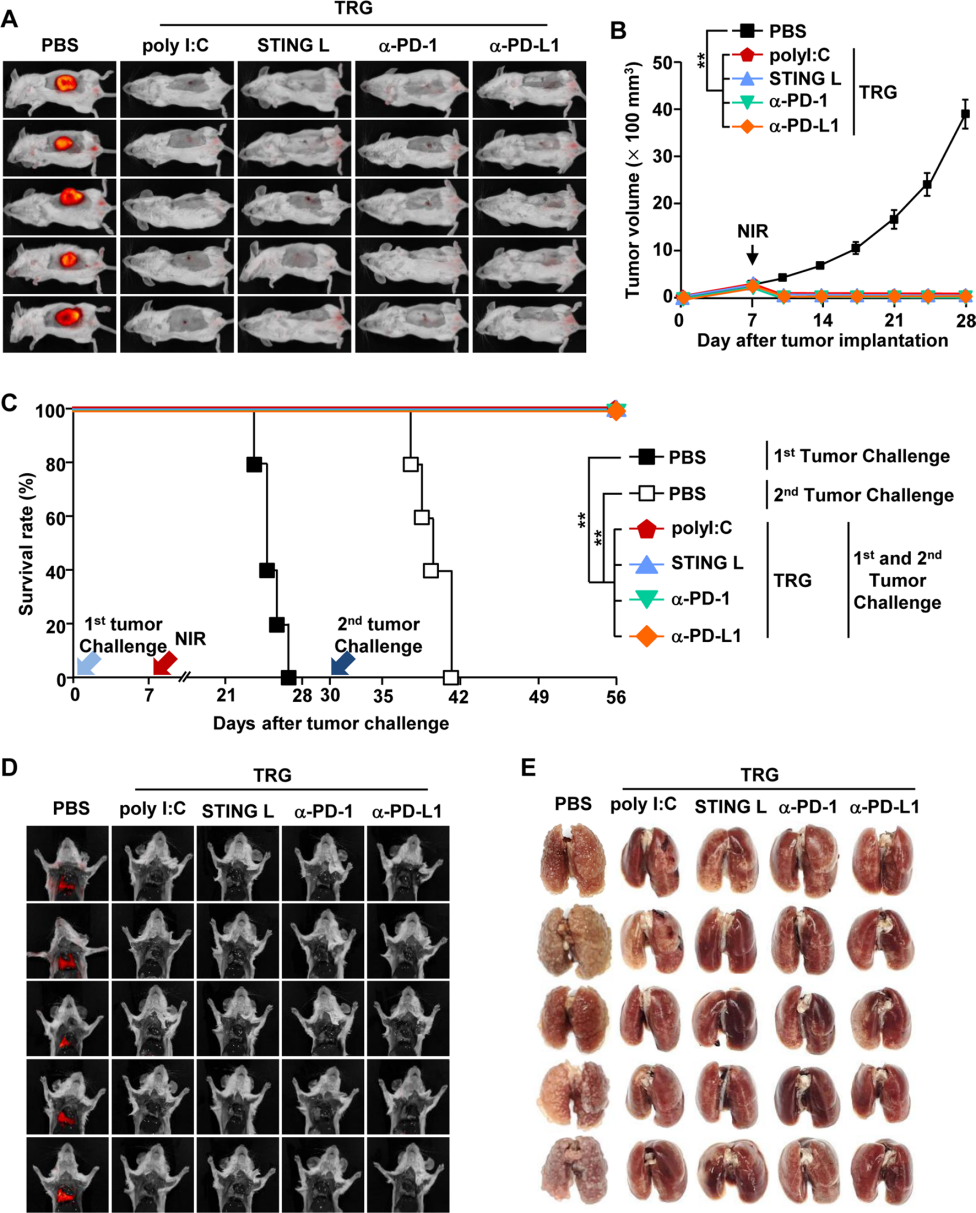
Role of TRG as a cancer therapeutic carrier for immunotherapy. On the 7th day after CT-26 tumor implantation, the mice were *i.t.* injected with TRGs containing either PBS, poly I:C, stimulator of interferon genes ligand (STING L), anti-PD-1 antibody (α-PD-1), or anti-PD-L1 antibody (α-PD-L1) under NIR laser irradiation (1.5 W/cm^2^, 5 min). **A** iRFP imaging in CT-26 tumor-bearing mice on the 20th day after tumor injection (n = 5). **B** Tumor growth curves of CT-26 mice (n = 6, significance was determined by the log-rank test, mean ± SEM **p < 0.01). **C** The survival rates of mice were monitored during the first and second challenges (n = 5 per group). **D** iRFP fluorescence images and **E** photographs of metastatic lung cancer 10 days after the second challenge (n = 5)

As the TRG can incorporate several types of molecules, we further examined whether it can act as a therapeutic carrier for the delivery of immunotherapeutic molecules to cancer cells. TRGs effectively released the incorporated immunostimulatory molecules at 60 °C, indicating that NIR irradiation induces the release of immunostimulatory molecules from TRGs (Additional file 1: Figure S4). Therefore, we next examined the effect of TRG as a therapeutic carrier, which was incorporated with poly I:C, STING ligand, anti-PD-1 Abs, and anti-PD-L1 Abs. CT-26 tumor-bearing mice were *i.t.* injected with these differently formulated TRGs and then irradiated with an NIR laser (Additional file 1: Figure S5). Laser irradiation in the immunotherapeutic molecule–containing TRGs did not alter the antibody activity (Additional file 1: Figure S6). Twenty days after primary tumor injection, the mice treated with the immunotherapeutic molecule–containing TRGs with NIR irradiation eliminated the first tumor (Fig. 7A, B). However, the mice treated with immunotherapeutic molecule–containing TRGs failed to inhibit tumor growth without NIR irradiation (Additioanl file 1: Figure S7). The cured mice from the first challenge were rechallenged with CT-26 cells via *i.v.* injection. These mice then survived the second tumor challenge 26 days later (Fig. 7C). In addition, TRGs containing poly I:C, STING ligand, anti-PD-1 Ab, or anti-PD-L1 Ab effectively eradicated the infiltrating CT-26 cancer cells in the lungs (Fig. 7D, E). Thus, the TRG can be used as a carrier of cancer therapeutic molecules, especially immunotherapeutic reagents.

## Discussion

Hydrogels offer the advantage of carrying various types of molecules [39]. In this study, we introduced ICG into our formulated hydrogel to induce a thermal response using an NIR laser. Moreover, we induced the release of poly I:C from the hydrogel, as high temperatures promoted its melting [40]. Intratumoral injection of pTRG followed by NIR laser irradiation promoted the apoptotic and necrotic death of CT-26 tumor cells, probably via the production of apoptotic bodies and tumor antigens [20, 41]. In addition to the apoptosis and necrosis of CT-26 cells induced by PTT, the incorporated poly I:C was released from pTRG. Therefore, poly I:C, together with CT-26 cell–associated antigen-stimulated DCs, elicited a T cell–mediated immune response. Consequently, these immune responses prevented the lung infiltration of CT-26 cells in mice after the second tumor challenge.

In the past, surgery, chemotherapy, and radiotherapy were the most common treatment methods for cancer [42]. However, these treatments are limited to metastatic cancers [2]. Moreover, surgical tumor removal cannot effectively prevent tumor recurrence [43]. With the establishment of immunotherapy, various treatment regimens have been combined with it to prevent cancer metastasis and recurrence [44]. In this study, we designed a combination therapy by treating the first transplanted tumor with PTT and preventing the second challenge of lung cancer metastasis via immunotherapy. The first transplanted tumor treated with PTT alone did not completely prevent the second challenge of tumor growth in the lungs. The treatment efficiency of the immune stimulators alone was almost nonexistent against both tumor challenges. However, the combination of PTT and immune activation by pTRG completely prevented the mice from developing tumors from the first and second tumor challenges, indicating their synergistic anticancer effects. The aim of PTT treatment was to eliminate the first transplanted tumor and to generate cancer-associated antigens. We found that the PTT-mediated tumor cell death led to the production of cancer antigens, and the release of poly I:C from TRG may have promoted cancer antigen–specific immune activation. This phenomenon consequently protected the mice from lung metastasis from the second tumor challenge.

PTT induces immunogenic cell death that stimulates immune cells via damage-associated molecular patterns (DAMPs) [45]. DAMPs released from dying cancer cells stimulate and recruit antigen-presenting cells [46]. Although several studies have shown that immunogenic cell death can effectively treat tumors by inducing immune activity, there are no studies that describe its effect in preventing cancer recurrence and metastasis [21, 45]. In this study, we found that the TRG-mediated cured mice from the first challenge with CT-26 tumors were also protected from lung metastasis from the second challenge. However, the preventive effect in these mice was not sufficient to prevent tumor growth caused by the second challenge, as shown in Fig. 4. Our previous study also showed that cured mice from the first tumor inoculated with PTT were only partially protected from the second challenge and eventually died due to cancer growth [20]. The reason for this failure in cancer protection may be that PTT killed both the cancer cells and the immune cells in the tumor microenvironment. These data indicate that anticancer immunity induced by immunogenic cell death is insufficient to prevent cancer recurrence or metastasis. Although PTT may have killed the DCs or macrophages in the tumor microenvironment, the DCs in tdLNs were effectively activated by the added immune stimulator instead of DAMPs. Since DAMPs secreted during immunogenic cell death are cancer cell-derived substances, we added an immune stimulator to elicit cancer antigen–specific immune activity. This prevented the mice from having lung tumor growth after the second cancer challenge. Therefore, the combination of cancer cell–associated DAMPs and immune stimulators can elicit an effective anticancer immunity.

Cancer treatment using hydrogels is being widely attempted, as hydrogels can effectively supply drugs. In a recent study, immunostimulatory molecules or immune checkpoint inhibitors were incorporated into hydrogels, which prevented cancer metastasis [47–50]. The hydrogel was applied by attaching it to the surgical site after the first transplanted tumor was surgically removed. This process effectively prevented the metastasis after the secondary challenge [47]. However, this is difficult to apply to patients because only 90% of the tumors are surgically removed to induce the expression of cancer antigens [47]. In this study, a method of killing primary cancer cells was applied to induce the expression of cancer antigens. Hydrogel-mediated PTT was applied to the first tumor to induce the expression of cancer antigens. Aside from the purpose of antigen release, our strategy showed perfect efficacy against the first challenge. In addition, the immunotherapeutic substances contained in the TRG were effectively released and eventually induced antigen-specific immune activity, which completely protected the mice from the secondary metastatic cancer challenge. Therefore, the TRG developed herein will be more efficient and effective for cancer treatment than the hydrogels used in previous studies.

In this study, we synthesized an injectable hydrogel that responds to an NIR laser to increase the temperature that allows the release of the incorporated molecules. The first tumor was eliminated via PTT, and the second challenge with lung metastatic cancer was prevented by immunotherapy induced by pTRG. In addition, the TRG can carry various immunotherapeutic molecules and effectively treat cancers. Thus, pTRG, which can be used as a carrier of immunotherapeutic reagents, is an efficient material that can treat primary tumors and prevent metastatic or recurrent cancers.

## Supplementary Information

**Additional file 1: Figure S1.** Thermographic images of CT-26 tumor-bearing mice intratumorally injected with PBS, poly I:C, Gel, pGel, TRG, and pTRG and then exposed to an 808-nm laser at a power density of 1.5 W/cm^2^ for 5 min (n = 6). **Figure S2.** Schematic illustration of the treatment strategy and the first and second tumor challenge models. **Figure S3.** Thermal images of 4T1 tumor-bearing mice intratumorally treated with PBS, poly I:C, Gel, TRG, and pTRG (left panel) and the average temperature after 808-nm laser irradiation (right panel, n = 6, ^**^p < 0.01). **Figure S4.** Cumulative release of the stimulator of interferon genes ligand (STING L), anti-PD-1 antibody (α-PD-1), and the anti-PD-L1 antibody (α-PD-L1) from TRGs after 808-nm laser irradiation (1.5 W/cm^2^, 5 min). **Figure S5.** Thermal images of CT-26 tumor-bearing mice irradiated with 808-nm laser (1.5 W/cm^2^, 5 min) after an *i.t.* injection of TRGs incorporated with poly I:C, stimulator of interferon genes ligand (STING L), anti-PD-1 antibody (α-PD-1), or anti-PD-L1 antibody (α-PD-L1). ** Figure S6**. Measurement of antibody activity after NIR irradiation in TRG. Anti-PD-1 and anti-PD-L1 antibodies were harvested from TRGs after NIR irradiation (1.5 W/cm^2^, 5 min). (A) Isolated CD3^+^ T cells were incubated with the released (A) anti-PD-1 antibodies and (B) anti-PD-L1 antibodies for 15 min, followed by secondary antibody (anti-goat-APC) staining for the evaluation of the functional activity of the released antibodies. **Figure S7**. Treatment with immune-stimulatory molecules failed to inhibit tumor growth. The CT-26 cells were injected into BALB/C mice as shown in Figure 7A. Indicated immune-stimulatory molecules containing TRGs were injected *i.t.* 7 days after tumor injection. (A) The representative tumor masses are shown 24 days after tumor injection. (B) CT-26 mouse tumor growth curves (n = 6).

## Data Availability

All data relevant to the study are included in the article or uploaded as online supplemental information.

## Abbreviations

PTT: Photothermal therapy
NIR: Near-infrared
ICG: Indocyanine green
DCs: Dendritic cells
MHC: Major histocompatibility complex
cDCs: Conventional dendritic cells
CTLs: Cytotoxic T lymphocytes
TRG: Thermally responsive hydrogel
poly I:C: Polyinosinic:polycytidylic acid
DW: Deionized water
STING: Stimulator of interferon genes
PD-1: Programmed cell death-1
PD-L1: Programmed cell death-ligand 1
PBS: Phosphate-buffered saline
FBS: Fetal bovine serum
*i.t.*: Intratumorally
Ab: Antibody
APC: Allophycocyanin
TCR: T cell receptor
PE: Phycoerythrin
IFN: Interferon
tdLN: Tumor-draining lymph node
*i.v.*: Intravenous
ELISA: Enzyme-linked immunosorbent assay
IL: Interleukin
TNF: Tumor necrosis factor
DAMP: Damage-associated molecular pattern
