# Supplementary material for: Dual-functional alginate and collagen–based injectable hydrogel for the treatment of cancer and its metastasis
Source: J Nanobiotechnology. 2022 May 28;20:245. doi: 10.1186/s12951-022-01458-x (PMC9148466; doi:10.1186/s12951-022-01458-x)
Supplement: Supplementary file 1 — Additional file 1: Figure S1. Thermographic images of CT-26 tumor-bearing mice intratumorally injected with PBS, poly I:C, Gel, pGel, TRG, and pTRG and then exposed to an 808-nm laser at a power density of 1.5 W/cm2 for 5 min (n = 6). Figure S2. Schematic illustration of the treatment strategy and the first and second tumor challenge models. Figure S3. Thermal images of 4T1 tumor-bearing mice intratumorally treated with PBS, poly I:C, Gel, TRG, and pTRG (left panel) and the average temperature after 808-nm laser irradiation (right panel, n = 6, **p < 0.01). Figure S4. Cumulative release of the stimulator of interferon genes ligand (STING L), anti-PD-1 antibody (α-PD-1), and the anti-PD-L1 antibody (α-PD-L1) from TRGs after 808-nm laser irradiation (1.5 W/cm2, 5 min). Figure S5. Thermal images of CT-26 tumor-bearing mice irradiated with 808-nm laser (1.5 W/cm2, 5 min) after an i.t. injection of TRGs incorporated with poly I:C, stimulator of interferon genes ligand (STING L), anti-PD-1 antibody (α-PD-1), or anti-PD-L1 antibody (α-PD-L1). Figure S6. Measurement of antibody activity after NIR irradiation in TRG. Anti-PD-1 and anti-PD-L1 antibodies were harvested from TRGs after NIR irradiation (1.5 W/cm2, 5 min). (A) Isolated CD3+ T cells were incubated with the released (A) anti-PD-1 antibodies and (B) anti-PD-L1 antibodies for 15 min, followed by secondary antibody (anti-goat-APC) staining for the evaluation of the functional activity of the released antibodies. Figure S7. Treatment with immune-stimulatory molecules failed to inhibit tumor growth. The CT-26 cells were injected into BALB/C mice as shown in Figure 7A. Indicated immune-stimulatory molecules containing TRGs were injected i.t. 7 days after tumor injection. (A) The representative tumor masses are shown 24 days after tumor injection. (B) CT-26 mouse tumor growth curves (n = 6). [file 12951_2022_1458_MOESM1_ESM.docx]

Supplemental Information

**Dual-functional alginate and collagen–based injectable hydrogel for the treatment of cancer and its metastasis**

Juyoung Hwang^1,2,3^, Eun-Koung An^2,3^, Wei Zhang^1^, Hyo Jeong Kim^4^, Youngho Eom^4^_,_ and Jun-O Jin^1, 2,3,^*

^1^ Shanghai Public Health Clinical Center & Institutes of Biomedical Sciences, Shanghai Medical College, Fudan University, Shanghai 201508, China

^2^ Department of Medical Biotechnology, Yeungnam University, Gyeongsan 38541, Republic of Korea

^3^ Research Institute of Cell Culture, Yeungnam University, Gyeongsan 38541, Republic of Korea

^4^ Department of Polymer Engineering, Pukyong National University, Busan 48513, Republic of Korea

*Corresponding author. E-mail: jinjo@yu.ac.kr

**
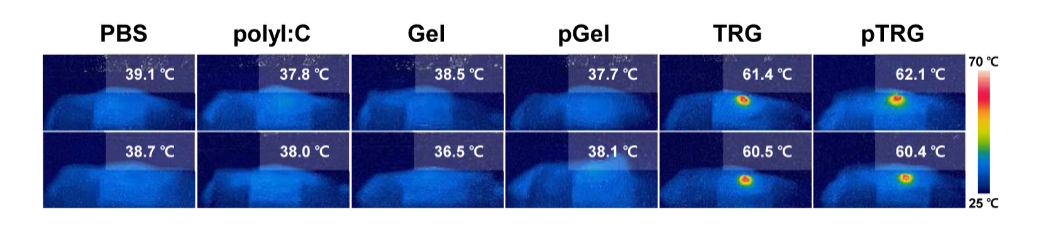
**

**Figure S1.** Thermographic images of CT-26 tumor-bearing mice intratumorally injected with PBS, poly I:C, Gel, pGel, TRG, and pTRG and then exposed to an 808-nm laser at a power density of 1.5 W/cm^2^ for 5 min (n = 6).


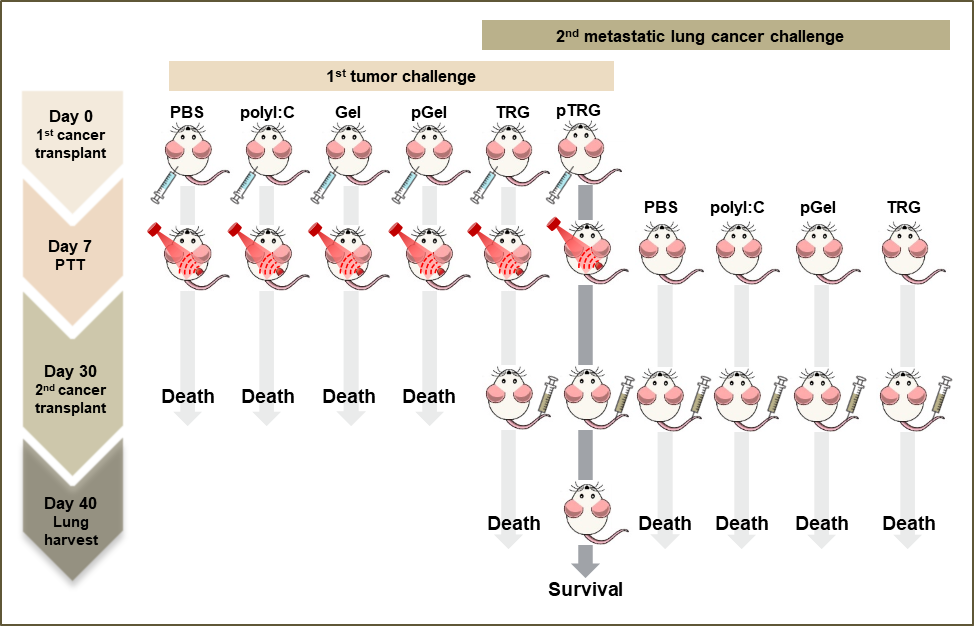


**Figure S2.** Schematic illustration of the treatment strategy and the first and second tumor challenge models


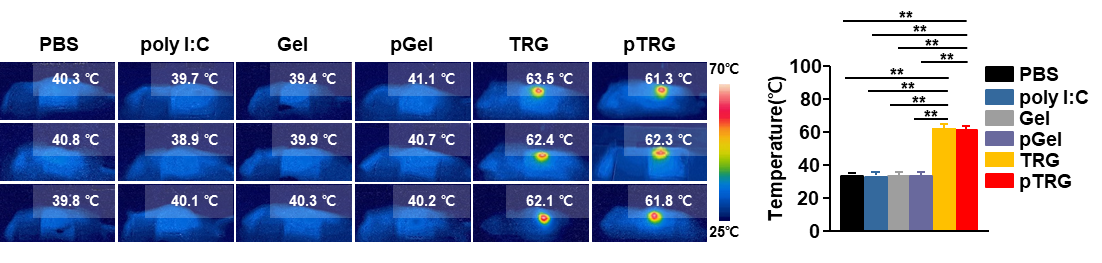


**Figure S3.** Thermal images of 4T1 tumor-bearing mice intratumorally treated with PBS, poly I:C, Gel, TRG, and pTRG (left panel) and the average temperature after 808-nm laser irradiation (right panel, n = 6, *^**^p* < 0.01)


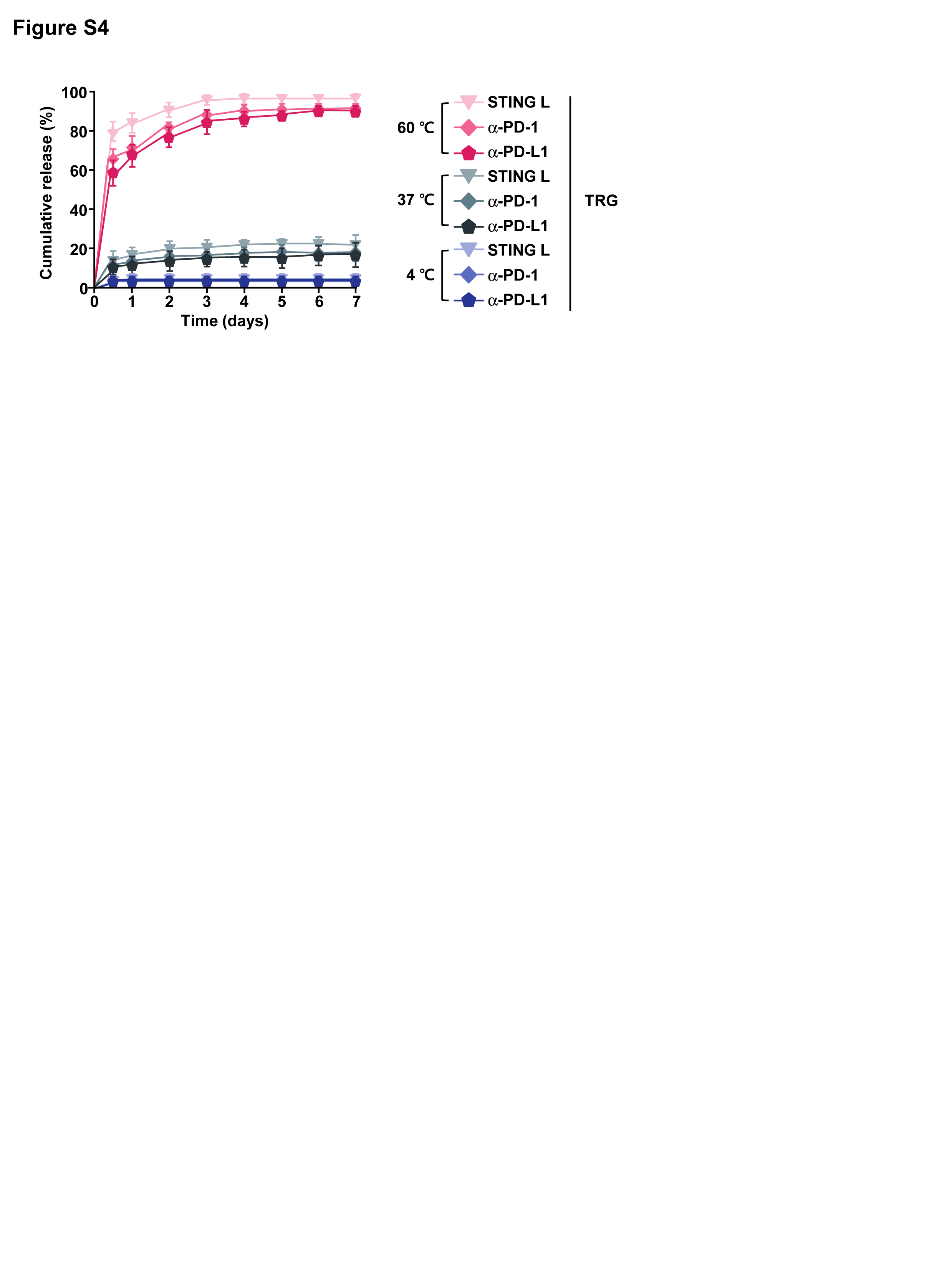


**Figure S4.** Cumulative release of the stimulator of interferon genes ligand (STING L), anti-PD-1 antibody (α-PD-1), and the anti-PD-L1 antibody (α-PD-L1) from TRGs after 808-nm laser irradiation (1.5 W/cm^2^, 5 min).


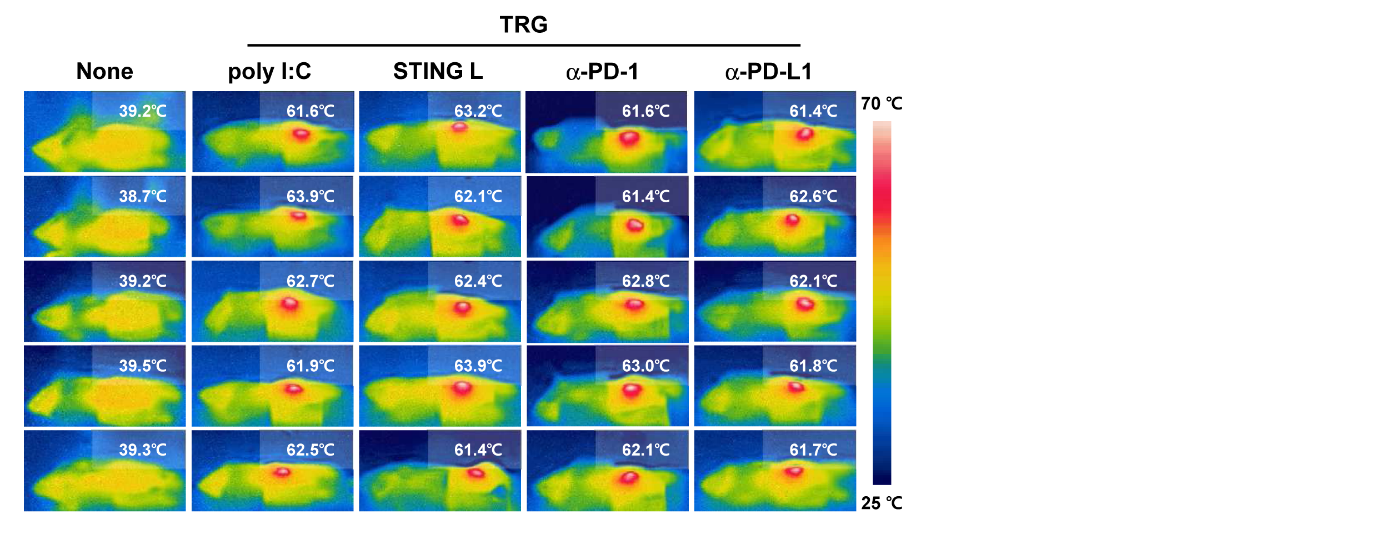


**Figure S5.** Thermal images of CT-26 tumor-bearing mice irradiated with 808-nm laser (1.5 W/cm^2^, 5 min) after an *i.t.* injection of TRGs incorporated with poly I:C, stimulator of interferon genes ligand (STING L), anti-PD-1 antibody (α-PD-1), or anti-PD-L1 antibody (α-PD-L1).

**
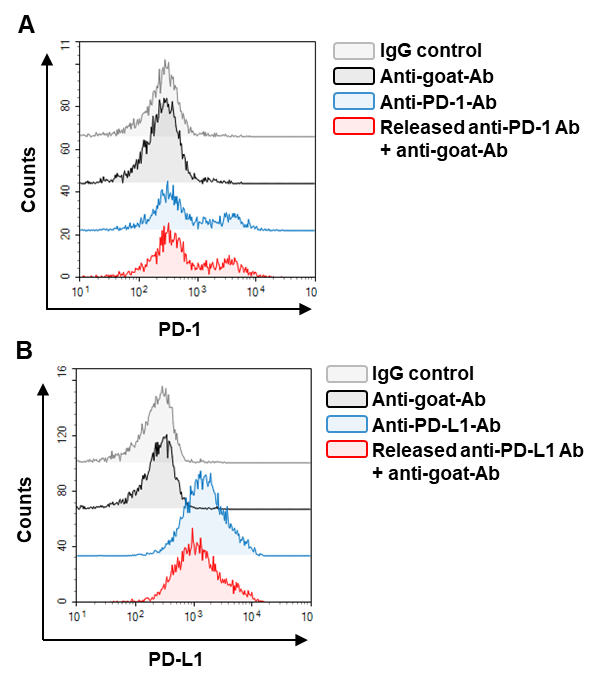
**

**Figure S6**. Measurement of antibody activity after NIR irradiation in TRG. Anti-PD-1 and anti-PD-L1 antibodies were harvested from TRGs after NIR irradiation (1.5 W/cm^2^, 5 min). (A) Isolated CD3^+^ T cells were incubated with the released (A) anti-PD-1 antibodies and (B) anti-PD-L1 antibodies for 15 min, followed by secondary antibody (anti-goat-APC) staining for the evaluation of the functional activity of the released antibodies.


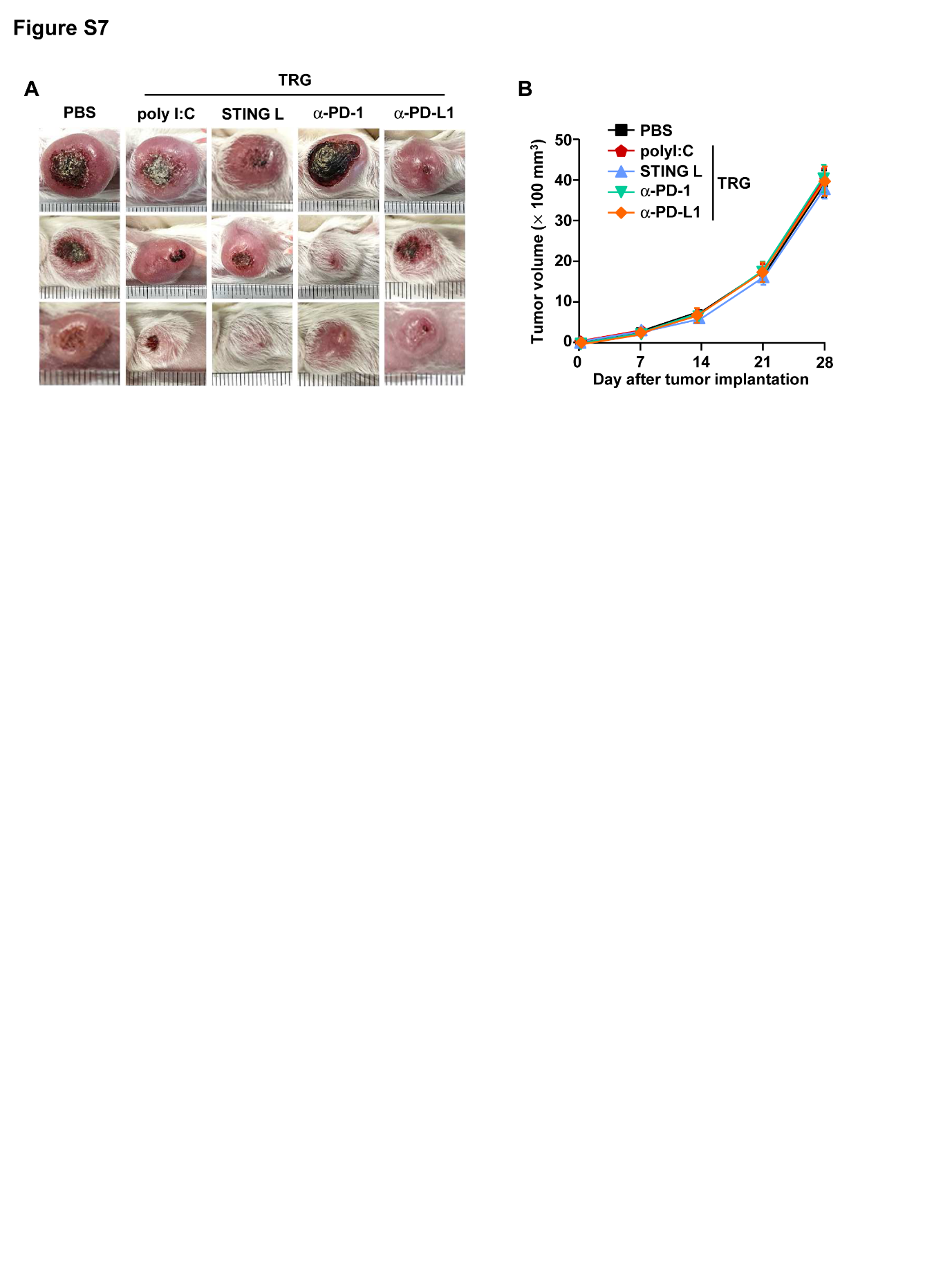
**Figure S7**. Treatment with immune-stimulatory molecules failed to inhibit tumor growth. The CT-26 cells were injected into BALB/C mice as shown in Figure 7A. Indicated immune-stimulatory molecules containing TRGs were injected *i.t.* 7 days after tumor injection. (A) The representative tumor masses are shown 24 days after tumor injection. (B) CT-26 mouse tumor growth curves (n = 6).
